# Reported bed net ownership and use in social contacts is associated with uptake of bed nets for malaria prevention in pregnant women in Ghana

**DOI:** 10.1186/s12936-016-1660-4

**Published:** 2017-01-04

**Authors:** Kacey C. Ernst, Steven Erly, Charity Adusei, Melanie L. Bell, David Komla Kessie, Alberta Biritwum-Nyarko, John Ehiri

**Affiliations:** 1 Department of Epidemiology and Biostatistics, Mel and Enid Zuckerman College of Public Health, University of Arizona, Campus PO Box 245211, 1295 N Martin Avenue, Tucson, AZ 85724 USA; 2 Kwame Nkrumah University of Science and Technology, Kumasi, Ghana; 3 Kumasi South Hospital, Kumasi, Ghana

**Keywords:** Malaria, Bed nets, Social influence, Interpersonal influence, Ghana, Prevention, Pregnancy

## Abstract

**Background:**

Despite progress made in the last decades, malaria persists as a pressing health issue in sub-Saharan Africa. Pregnant women are particularly vulnerable to infection and serious health outcomes for themselves and their unborn child. Risk can be mitigated through appropriate use of control measures such as insecticide-treated bed nets. Although social networks can influence uptake of preventive strategies, the role of social influence on bed net ownership has not been explored. During an evaluation of a bed net distribution programme, the influence of non-health care advisors on ownership and use of bed nets by pregnant women in Kumasi, Ghana was examined.

**Methods:**

Data were collected through in-person interviews with 300 pregnant women seeking antenatal care in an urban hospital in Kumasi, Ghana. Participants were asked about their bed net ownership, bed net use, and information about three personal contacts that they go to for pregnancy advice. Information about these advisors was combined into an influence score. Logistic regression models were used to determine the association between the score and bed net ownership. Those who owned a bed net were further assessed to determine if interpersonal influence was associated with self-reported sleeping under the bed net the previous night.

**Results:**

Of the 294 women in the analysis, 229 (78%) reported owning bed nets. Of these bed net owners, 139 (61%) reported using a bed net the previous night. A dose response relationship was observed between the interpersonal influence score and bed net ownership and use. Compared to the lowest influence score, those with the highest influence score (>1 SD above the mean) were marginally more likely to own a bed net [OR = 2.37, 95% CI (0.87, 6.39)] and much more likely to use their bed net [5.38, 95% CI (1.89, 15.25)] after adjusting for other factors.

**Conclusions:**

Interpersonal influence appears to have modest impact on ownership and use of bed nets by pregnant women in an urban area of Ghana. Further investigations would need to be conducted to determine if the relationship is causal or if individuals who associate are simply more likely to have similar practices.

**Electronic supplementary material:**

The online version of this article (doi:10.1186/s12936-016-1660-4) contains supplementary material, which is available to authorized users.

## Background

Since the turn of the century the number of malaria deaths has been reduced by nearly half [1]. This progress in the fight against malaria has been made in part due to the increased distribution and use of long-lasting insecticidal nets (LLINs). LLIN have proven to be one of the most effective tools to combat the disease, providing both individual and communal benefits [1–3]. Mass distribution campaigns and clinic-based distributions are the most commonly used strategies to deliver LLIN to communities, yet ownership and usage rates remain well below the targeted ratio of 1 bed net per 2 persons [4]. This shortcoming is especially pronounced in vulnerable and at-risk populations, notably children and pregnant women [5–7].

Adoption of LLIN usage is particularly important for pregnant women. Pregnant women are more vulnerable to malaria and severe outcomes for multiple reasons: enhanced attractiveness to the mosquito due to increased respiratory volume and skin temperature, suppression of the immune system and the sequestration of the malaria parasite in the placenta [8, 9]. The unborn child is subsequently at increased risk for spontaneous abortion, stillbirth, low birthweight, and prematurity [2]. Despite the increased risks and the prioritization of pregnant women in ITN campaigns, bed net usage among pregnant women in malaria transmission zones has plateaued in the recent years and ranges from as low as 16% (Nigeria) to 74% (Benin) across sub-Saharan African countries that have high rates of malaria transmission. Most countries hover around 50% [1, 10, 11].

Malaria in Ghana is still highly endemic, an estimated 580,000–11,000,000 cases of malaria occurred in 2013 and the entire country is classified as high transmission (>1 case per 1000 population). Free distribution of ITN/LLIN was instituted in 2004, and large-scale roll-outs of bed nets have been conducted since 2007 with expansion to all age groups in 2010 [12, 13]. Antenatal care-based distributions have been established as a key strategy to improve access to bed nets for pregnant women, although gaps in coverage persist [14]. In 2011, only 35% of pregnant women between the ages of 15 and 49 in the Ghana Demographic and Health Survey reported sleeping under a bed net and even fewer (32%) slept under a LLIN the night prior to survey [15]. New approaches to understand these gaps and to reach the universal coverage goals laid out by the Roll Back Malaria Partnership are needed.

While the body of literature has grown on social and communal factors affecting bed net use, little has been published on the role of interpersonal influences in the uptake of bed nets [16, 17]. Interpersonal influence, which is comprised of social support, social norms, and role models, is a key component of the health promotion model, and could be expected to be a factor in the decision to own or use a bed net [18]. Interpersonal influences have been found to be critically important in the adoption of health promoting behaviors in other contexts [18–22]. The larger concept of ideation, which includes beliefs and values, social norms, emotional responses, and social support and influence about a particular subject has been demonstrated to be correlated with household ITN use in Tanzania [23].

Understanding the role of interpersonal influence on the decision to obtain or use bed nets could lead to improvements in their promotion. The primary objective of this study, therefore, was to determine whether interpersonal influences were associated with ownership and/or use of bed nets in pregnant women attending antenatal care in Kumasi, Ghana. Simple measures of interpersonal influence were collected for self-reported advisors to pregnant women in Kumasi, Ghana, and modelled into a single influence score. This influence score was then related to the bed net practices of the pregnant women themselves.

## Methods

### Study setting and design

Cross sectional data were collected from pregnant women seeking antenatal care at Kumasi South Hospital in Kumasi, Ghana. Data were collected as part of a wider survey investigating bed net use and malaria prevention strategies among pregnant women in 2014. All questions were translated by locals to ensure cultural compatibility and acceptability.

Kumasi South Hospital (KSH) is a primary care hospital in south-central Ghana [24]. KSH is located between three cities (Atonsu, Agogo and Chirapatre) in the Ashanti Region of Ghana and provides services to 56 communities which consist of approximately 400,000 people [25]. Kumasi, along with the rest of Ghana, is considered a high-malaria transmission zone by the World Health Organization (WHO), though the prevalence of malaria in urban areas in Ghana is significantly lower than in equivalent rural communities [26–28]. There have been mass distribution campaigns to increase bed net usage in the Ashanti region including subsidization of bed net purchases until 2010 and then door-to-door distribution of bed nets through 2012 [15]. According to the 2014 Ghana Demographic and Health Survey, 86.2% of bed nets in the Ashanti Region were acquired for free and 82.3% came from the public sector, although this information may have been collected after the study survey was administered [29].

In March 2014, which represents the beginning of the first rainy season in Kumasi, a systematic sample of women attending the clinic for antenatal care were identified and interviewed on-site. Women were selected by retrieving the last file in the stack of charts that were placed in order of appointment on the desk of the hospital from clinic open to close, Monday–Friday. After an interview was conducted another chart was selected. This process minimized bias by selecting women throughout the day and week. In addition, selecting the chart from the bottom of the pile provided enough time between selection and clinic appointment to conduct the interview. The in-person survey was conducted with trained personnel to determine knowledge, attitudes and practices surrounding bed nets and other malaria prevention strategies.

### Variables

The exposure of interest was interpersonal influence. This was determined by asking participants to provide information for up to three advisors outside of medical personnel who they sought advice from during pregnancy. For each self-reported advisor, the women were asked whether the advisor uses a bed net, whether the advisor had talked with the participant about malaria, and how often the participant followed the advisor’s advice. The advisors were also ranked by whom the women went to first, second and third. The advisor score, for each participant, was transformed into a single score with the formula:

$${\text{Influence Score}} = \mathop \sum \limits_{j = 1}^{J} R_{j} *\left( {U_{j} + T_{j} } \right)*I_{j}$$

where J = the number of advisors; R_j_ = Weight of the jth advisor’s influence by rank (1st advisor received a weight of 1, 2nd advisor received a weight of 0.75, 3rd advisor received a weight of 0.5); U_J_ = Bed net use by the jth advisor (1 for yes, 0 for don’t know, −1 for no); T_j_ = Talk with jth advisor about malaria during pregnancy (1 for yes, 0 for no/don’t know); I_j_ = How often the jth advisor’s advice is followed (1 for very often, 0.75 for sometimes, 0.5 for never).

Influence scores could range from 4.5 for a participant who received strong interpersonal influence towards using a bed net to −2.25 for a participant who received no interpersonal influence towards using a bed net.

The outcomes of interest were self-reported ownership and use of a bed net. Given there were still some untreated bed nets in circulation at the time, all bed nets (LLIN, insecticide-treated nets, or untreated nets) were treated equally and dichotomized into use/non-use and ownership/non-ownership. Bed net use was assigned on reported use the night prior to the survey, per WHO guidelines [30, 31]. In addition, another question was asked about bed net use to conduct a sensitivity analysis, namely, “Do you use a mosquito net?” The bed net ownership analysis was performed on all of the women sampled, while the bed net use analysis was performed only on the subset of the women that owned a bed net.

Other variables included in the analyses were the participants’ ages, education levels, marital statuses, malaria perceptions and knowledge, and perceived availability of bed nets in the community. Age, education level, and marital status were included on face validity. Perceptions and knowledge of a disease are crucial aspects of the health promotion model and are likely associated with bed net ownership and use. They were included in the adjusted models as potential explanatory factors of the association between interpersonal influence and bed net ownership and use [22]. Aspects of the perceived risk of malaria, perceived severity of malaria, and knowledge of malaria prevention were determined using the questions listed in Table 1. Each question was treated as an individual variable. Perceived bed net availability was included in the adjusted models, as availability of bed nets in the community may confound the relationship between influence score (advisor use of bed nets being dependent on availability) and bed net ownership by the participants. Perceived availability was measured by the question, “Is it easy to obtain a mosquito net during pregnancy?”

**Table 1:** Characteristics of 294 pregnant women seeking antenatal care in Kumasi, Ghana

Characteristic	All women (n = 294)	Bed net owner (n = 227)	Bed net non-owner (n = 67)	Bed net user (n = 137)	Bed net non-user (n = 90)
Age (years), mean (SD)	26.7 (5.65)	27.0 (5.6)	25.7 (5.8)	27.5 (5.3)	26.2 (5.9)
Marital status, n (%)					
Married	159 (54.1)	126 (55.5)	33 (49.3)	84 (61.3)*	42 (46.7)
Unmarried	135 (45.9)	101 (44.5)	34 (50.7)	53 (38.7)	48 (53.3)
Education level, n (%)					
None/primary/don’t know	57 (19.4)	38 (16.7)	19 (28.4)	28 (20.4)	10 (11.1)
JSS/JHS	137 (46.6)	108 (47.6)	29 (43.3)	61 (44.5)	47 (52.2)
SSS, SHS, vocational or tertiary	100 (34.0)	81 (35.7)	19 (28.4)	48 (35.0)	33 (36.7)
Malaria attitudes and perceptions, n (%)					
One or more advisors got malaria when they were pregnant	43 (14.6)	29 (12.8)	14 (20.9)	14 (10.22)	15 (16.7)
Is worried about getting malaria	215 (73.1)	171 (75.3)	44 (65.7)	106 (77.4)	65 (72.2)
Has heard about malaria in the last year	234 (80.0)	183 (80.6)	47 (70.2)	116 (84.7)	67 (74.4)
Knows someone who died from malaria	40 (13.6)	34 (15.0)	6 (9.0)	20 (14.6)	14 (15.6)
Has heard about using mosquito nets	281 (95.6)	217 (95.6)	64 (95.5)	131 (95.6)	86 (95.6)
Availability of bed nets, n (%)					
It is easy to obtain a free mosquito net during your pregnancy					
Yes	218 (74.1)	175 (77.1)*	43 (64.2)	111 (81.0)	64 (71.1)
No/unsure	76 (25.9)	52 (22.9)	24 (35.8)	26 (19.0)	26 (28.9)
Number of advisors, n (%)					
0	21 (7.1)	19 (8.3)	2 (3.0)	14 (10.2)	5 (5.6)
1	36 (12.2)	28 (12.3)	8 (11.9)	18 (13.4)	10 (11.1)
2	52 (17.7)	42 (18.5)	10 (14.9)	29 (21.2)	13 (14.4)
3	185 (61.9)	138 (60.7)	47 (70.1)	76 (55.5)	62 (68.9)
Influence Score^a^, mean (SD)	1.15 (1.67)	1.23 (1.67)	0.85 (1.66)	1.51 (1.61)*	0.80 (1.67)

* p < 0.05

^a^ $${\text{Influence Score}} = \sum\nolimits_{{{\text{n}} = 1}}^{\text{j}} {{\text{R}}_{\text{j}} *\left( {{\text{U}}_{\text{j}} + {\text{T}}_{\text{j}} } \right) * {\text{I}}_{\text{j}} }$$

### Analysis

A preliminary assessment of the population was performed using the measured and derived variables. Two-sample t tests for the continuous variables and Chi square tests for the categorical variables were used to detect differences across the outcomes (bed net ownership and use.) Categorical variables were assessed for sparse data. Variables with categories that contained less than 5% of the total number of observations were excluded from the model or recategorized.

The primary analysis consisted of a logistic regression model between influence score and the binary outcomes, bed net ownership and bed net use. For each outcome, three models were constructed: A crude model with only the influence score, “Adjusted A” with influence score, age, marital status, and education level, and “Adjusted B” with the perceived availability of bed nets and malaria perception and knowledge variables in Table 1 in addition to the variables in Adjusted A. Odds ratios for the influence scores were calculated as the measure of association. Area under the receiver operating characteristic curve (AUC) values were calculated to determine the ability of the influence score to predict bed net ownership and use, i.e., model fit.

Age and influence score were assessed for linearity against the logit of the probability of the outcomes and were transformed or categorized if linearity was not observed. Observations with missing values for any variable were excluded from the analysis. All analysis was performed using SAS 9.4.

### Sensitivity analyses

The method of aggregation of the variables into the influence score was tested by comparing the score outlined above with two alternative scoring systems; an influence score unweighted by advisor ranking, [${\text{Influence Score}} = \sum\nolimits_{{{\text{j}} = 1}}^{\text{j}} {\left( {{\text{U}}_{\text{j}} + {\text{T}}_{\text{J}} } \right) * {\text{I}}_{\text{J}} }$], and a simple sum of the number of reported advisors who used bed nets [${\text{Influence Score}} = \sum\nolimits_{{{\text{j}} = 1}}^{\text{j}} {({\text{U}}_{\text{j}} )}$]. The odds ratios of the influence scores, significance of the influence scores, and AUC values of new models were compared to the original.

In addition, comparisons were made between the WHO recommended method of determining bed net usage (“Last night did you sleep under this net?”) and an alternative question regarding overall bed net use (“Do you use a mosquito net?”). The net use model using this new question was compared to the original. A further model that assigned bed net use only when a participant answered “yes” to both questions was also compared to the original.

## Results

Of the 300 women sampled, 294 (98%) provided complete survey information and were included in the analysis. The six women not included did not provide information about their advisors during pregnancy. Of the 294 women included in analysis, 229 (78%) reported owning bed nets. Of these bed net owners, 139 (61%) reported having used a bed net the previous night for an overall estimate of 47% of pregnant women sleeping under a bed net.

Age, education level, and malaria knowledge and perceptions were similar across all outcomes. A significantly higher (p = 0.024) proportion of net users were married as compared to non-net users, though no difference was detected between net owners and non-net owners (p = 0.442). Bed net owners were more likely to perceive bed nets as easy to get than non-bed net owners (p = 0.035), though no difference was detected between net users and non-users (p = 0.724).

Influence scores differed across the outcome of bed net ownership, though not significantly. Bed net owners had a mean score of 1.23 [standard deviation (SD) = 1.67] and non-owners had a mean score of 0.85 (SD = 1.66, p = 0.094). Across bed net use, the difference was significant. Bed net users had a mean score of 1.51 (SD = 1.61), while non-users had a mean score of 0.80 (SD = 1.67, p = 0.001) (Table 1).

### Advisor characteristics

There were 692 advisors of the pregnant women in the sample. Among the advisors whose gender could be determined from the information in the survey, the majority were female (56.8%). The average advisor age reported by the pregnant women was 43.5 (SD = 16.4). The most common advisors were the pregnant woman’s mother (30.3%), the pregnant woman’s sister (15.4%), and the pregnant woman’s husband (13.1%). There was a significant difference in gender categories between the advisors of bed net users and non-users (p = 0.0469). No other differences were found in age or relationship across either category or gender across bed net ownership.

There were differences in advisor characteristics across outcomes, with a significantly higher proportion of the advisors of net owners (52.8%) using bed nets than the advisors of non-owners (38.9%, p = 0.0004). A similar difference was seen across net use categories (61.4 and 40.1% for users and non-users, p < 0.0001). The frequencies of having had discussions about malaria during pregnancy and of following advice were not significantly different across outcomes for ownership groups (p = 0.0617, and p = 0.5722, respectively) or use groups (p = 0.6950 and p = 0.1348).

The differences seen in influence score across the outcomes for the pregnant women was mirrored in their advisors. Advisors of bed net owners had higher mean influence score than advisors of non-bed net owners but not significantly (bed net owners: mean = 0.41, SD = 0.62. Non bed net owners: mean = 0.32, SD = 0.57, p = 0.0963). The mean influence score for advisors of net users (0.52, SD = 0.57) was significantly higher than those of non-net users was (0.26, SD = 0.65, p = 0.001) (Table 2).

**Table 2:** Demographic and influence characteristics of advisors of pregnant women seeking antenatal care in Kumasi, Ghana

Characteristic	All advisors (n = 692)	Advisor of bed net owner (n = 525)	Advisor of bed net non-owner (n = 167)	Advisor of bed net user (n = 303)	Advisor of bed net non-user (n = 222)	Scoring
Age (years), mean (SD)	43.5 (16.4)	43.9 (16.4)	42.4 (16.2)	43.5 (16.0)	44.4 (17.0)	N/A
Gender, n (%)						
Male	141 (20.4)	107 (20.3)	34 (20.4)	76 (25.1)	36 (16.2)	N/A
Female	393 (56.8)	307 (58.5)	86 (51.5)	170 (56.1)	142 (64.0)	N/A
Unknown	158 (22.8)	111 (21.1)	47 (28.1)	57 (18.8)	44 (19.8)	N/A
Relationship, n (%)						
Mother	210 (30.3)	165 (31.4)	45 (26.9)	98 (32.3)	66 (29.7)	N/A
Sister	107 (15.4)	85 (16.2)	22 (13.1)	44 (14.5)	41 (18.5)	N/A
Husband	90 (13.0)	69 (13.1)	21 (12.6)	43 (14.2)	26 (11.7)	N/A
Aunt	23 (3.3)	12 (2.3)	11 (6.6)	6 (2.0)	6 (2.7)	N/A
Friend	75 (10.8)	52 (9.9)	23 (13.8)	31 (10.2)	22 (9.9)	N/A
Other	187 (27.0)	142 (27.0)	45 (26.9)	81 (26.7)	61 (27.5)	NA
Uses a bed net (U), n (%)						
Yes	342 (49.4)	277 (52.8)*	65 (38.9)	186 (61.4)*	91 (41.0)	1
No	241 (34.8)	162 (30.9)	79 (47.3)	64 (21.1)	98 (44.1)	−1
Don’t know	109 (15.8)	86 (16.4)	23 (13.8)	53 (17.5)	33 (14.9)	0
How often is advice followed (I), n (%)						
Very often	448 (64.7)	337 (64.2)	111 (66.5)	190 (62.7)	147 (66.2)	1
Sometimes	221 (31.9)	172 (32.8)	49 (29.3)	103 (34.0)	69 (31.1)	0.75
Never	23 (3.3)	16 (3.1)	7 (4.2)	10 (3.3)	6 (2.7)	0.5
Talked about malaria during pregnancy (T), n (%)						
Yes	365 (52.7)	266 (50.7)	99 (59.3)	162 (53.5)	104 (46.9)	1
No/don’t know	327 (47.3)	259 (49.3)	68 (40.7)	141 (46.5)	118 (53.2)	0
Influence Score, mean (SD)	0.35 (6.1)	0.41 (0.62)	0.32 (0.57)	0.52 (0.57)*	0.26 (0.65)	a

* p < 0.05

^a^ $${\text{Influence Score}} = \sum\nolimits_{{{\text{j}} = 1}}^{\text{j}} {{\text{R}}_{\text{j}} *\left( {{\text{U}}_{\text{j}} + {\text{T}}_{\text{j}} } \right) * {\text{I}}_{\text{j}} }$$

### Models

Age and influence score were found to be nonlinear with respect to the logit of the probability of bed net ownership and were categorized. Age was categorized into quartiles. The influence score was categorized into standard deviations from the mean to maximize the interpretability of the findings. Crude and adjusted logistic regression models were constructed, and odds ratios were calculated as the odds of using or owning a net with the lowest categories of influence score and age as references (see Additional files 1, 2).

The results of the crude, Adjusted A, and Adjusted B models were similar (Table 3). Participants with a higher influence score were more likely to own a bed net. In the Adjusted B model, as compared to the reference category (>1 SD below the mean,) participants with an influence score within one SD below the mean had an odds ratio of 1.39 [95% CI (0.61, 3.13)], participants with an influence score within one SD above the mean had an odds ratio of 2.89 [95% CI (1.13, 7.39)], and participants with an influence score greater than one SD above the mean had an odds ratio of 2.37 [95% CI (0.88, 6.39)]. The area under the ROC curve of the crude bed net ownership model was 0.588.

**Table 3:** Odds ratios of reported bed net ownership and use by standard deviation of Influence Score^a^

Influence Score^a^	Crude	Adjusted A^b^	Adjusted B^c^
	OR (95% CI)	OR (95% CI)	OR (95% CI)
Bed net ownership (n = 294)			
>1 SD below mean	Reference	Reference	Reference
0–1 SD below mean	1.36 (0.65, 2.85)	1.27 (0.58, 2.77)	1.39 (0.61, 3.13)
0–1 SD above mean	2.46 (1.03, 5.88)	2.74 (1.11, 6.75)	2.89 (1.13, 7.49)
>1 SD above mean	2.11 (0.84, 5.29)	2.49 (0.95, 6.54)	2.37 (0.87, 6.39)
Model AUC	0.588	0.685	0.725
Bed net use (n = 227)			
>1 SD below mean	Reference	Reference	Reference
0–1 SD below mean	2.80 (1.23, 6.40)	2.35 (1.00, 5.53)	2.28 (0.93, 5.56)
0–1 SD above mean	3.19 (1.35, 7.61)	3.01 (1.23, 7.34)	2.76 (1.10, 6.94)
>1 SD above mean	5.50 (2.06, 14.65)	5.25 (1.92, 14.39)	5.38 (1.89, 15.25)
Model AUC	0.620	0.664	0.707

^a^ $${\text{Influence Score}} = \sum\nolimits_{{{\text{j}} = 1}}^{\text{j}} {{\text{R}}_{\text{j}} *\left( {{\text{U}}_{\text{j}} + {\text{T}}_{\text{j}} } \right) * {\text{I}}_{\text{j}} }$$

^b^Adjusted for age, marital status, and education level

^c^Adjusted for age, marital status, education level, and malaria perceptions and attitudes

The same trend was seen in net use. Using the lowest category of influence score (>1 SD below mean) as the reference category, participants with an influence score within one SD below the mean had an odds ratio of 2.28 [95% CI (0.93, 5.56)], participants with an influence score within one SD above the mean had an odds ratio of 2.76 [95% CI (1.10, 6.94)], and participants with an influence score greater than one SD above the mean had an odds ratio of 5.38 [95% CI (1.89, 15.25)] in the Adjusted B model (Table 3). The area under the ROC curve of the crude bed net use model was 0.620.

### Sensitivity analyses

Separate analyses were performed using the alternative influence score formulas: the original influence score unweighted by advisor rank [${\text{Influence Score}} = \sum\nolimits_{{{\text{j}} = 1}}^{\text{j}} {\left( {{\text{U}}_{\text{j}} + {\text{T}}_{\text{J}} } \right) * {\text{I}}_{\text{J}} }$] and the number of advisors using nets [${\text{Influence Score}} = \sum\nolimits_{{{\text{j}} = 1}}^{\text{j}} {({\text{U}}_{\text{j}} )}$]. The analysis using the influence score unweighted by advisor rank yielded odds ratios and areas under the receiver operating characteristic curve similar to the primary models (see Additional file 3). Notable was the similarity of the analysis that used solely the number of advisors using bed nets to calculate the influence score. This analysis yielded similar trends in the odds ratios, significance of the exposure terms, and AUC values to the primary models. As the number of advisors using bed nets increased, the odds ratios for bed net ownership and use also increased. With 0 advisors using a bed net as the reference category, the odds of owning a bed net were 1.12 times higher with 1 advisor [95% CI (0.55, 2.30)], 1.98 times higher with 2 advisors [95% CI (0.86, 4.53)], and 2.45 times higher with 3 advisors [95% CI (0.91, 6.58)]. The odds of using a bed net were 0.94 times higher with 1 advisor [95% CI (0.46, 1.94)], 1.88 times higher with 2 advisors [95% CI (0.85, 4.18)] and 4.29 times higher with 3 advisors [95% CI (1.54, 11.96)]. The areas under the ROC curves were 0.563 for the crude ownership model and 0.590 for the crude use model (Table 4). No relationship was seen between the other terms of the influence score and the outcomes (see Additional file 3).

**Table 4:** Odds ratios of reported bed net ownership and use by number of advisors using nets

Number of advisors using nets	Crude	Adjusted A^a^	Adjusted B^b^
	OR (95% CI)	OR (95% CI)	OR (95% CI)
Bed net ownership (n = 294)			
0	Reference	Reference	Reference
1	1.15 (0.59, 2.22)	1.17 (0.59, 2.32)	1.21 (0.55, 2.30)
2	1.67 (0.78, 3.58)	1.89 (0.85, 4.20)	1.98 (0.86, 4.53)
3	1.85 (0.73, 4.46)	2.36 (0.90, 6.19)	2.45 (0.91, 6.58)
Model AUC	0.563	0.671	0.715
Bed net use (n = 227)			
0	Reference	Reference	Reference
1	1.08 (0.55, 2.12)	1.08 (0.54, 2.17)	0.94 (0.46, 1.94)
2	1.61 (0.78, 3.31)	1.94 (0.91, 4.17)	1.88 (0.85, 4.19)
3	2.94 (1.18, 7.33)	3.52 (1.34, 9.28)	4.29 (1.54,11.96)
Model AUC	0.590	0.662	0.713

^a^Adjusted for age, marital status, and education level

^b^Adjusted for age, marital status, education level, and malaria perceptions and attitudes

The more general question about bed net use, “Do you use a mosquito net?” included a larger percentage of women in the bed net use category than did the original WHO question (69 vs 60%). The more sensitive definition of bed net use was associated with the influence terms at a level several-fold higher than the WHO definition. For the most adjusted model, using the lowest category of influence score (>1 SD below mean) as the reference category, participants with an influence score within one SD below the mean had an odds ratio of 2.78 [95% CI (1.13, 6.89)], participants with an influence score within one SD above the mean had an odds ratio of 5.57 [95% CI (2.08, 14.84)], and participants with an influence score greater than one SD above the mean had an odds ratio of 10.91 [95% CI (3.35, 35.56)]. The results of these models suggests that the WHO recommended question for bed net use is appropriate as a conservative measure of bed net usage.

## Discussion

Pregnant women reporting to Kumasi South Hospital for antenatal care that owned and used bed nets were more likely to have a higher reported number of advisors that also used bed nets. A dose–response was seen between the influence score and both bed net ownership and bed net use. This analysis suggests that when a pregnant woman receives advice from a social network that includes other individuals who use bed nets it may provide cues for the pregnant woman to increase the ownership and use of bed nets. The AUC values indicate, however, that there were additional unmeasured factors that must be included to fully explain gaps in bed net ownership and use.

Ownership was notably not as strongly related to the number of advisors that used bed nets as use. This may in part be due to the fundamentally different role that an advisor can play in the attainment of a bed net as compared to the use of an already owned bed net. As indicated previously, most bed nets are acquired through free distribution networks. A pregnant woman’s ability to acquire a bed net is impacted significantly by availability at the clinic where stock-outs and interruptions to the supply chain have influenced the level of ownership by pregnant women [7]. The use of owned bed nets is less sensitive to fluctuations in availability and other external factors and may explain the discrepancy in the association between the influence of advisors on ownership as compared to use. While a significant body of research focuses on the social and behavioral influences of bed net use, the social factors investigated are often education and income status [17, 32–34]. No other studies specifically addressing the role of interpersonal relationships or direct peer influence and bed net use were identified in the published literature during the course of the study. However, exposure to community change agents (CCA), educators who are from and work within communities to actively engage families about malaria prevention and control, were found to have modestly influenced household-level universal coverage targets through its influence on net ideation [23]. There is, however, relative consistency in the associations between social factors such as education, income and bed net ownership and use among both pregnant women and the general population [7, 17, 22]. Further investigation into the associations between interpersonal influence on bed net ownership and use should be undertaken in a larger and more diverse sample to determine the robustness and generalizability of the findings.

Though these findings are unique when examining determinants of bed net ownership and use, the influence of interpersonal relationships on health behaviors is not without precedent. It is well-established that both peers and family members influence the uptake of both healthy and unhealthy habits [19, 35, 36]. In the realm of women’s reproductive health, a systematic review of factors associated with accessing antenatal care identified the importance of social support [37]. A qualitative study in three countries in sub-Saharan Africa indicates that the support of friends and relatives is influential in seeking antenatal care particularly if they have a role in healthcare [38]. In Ghana, antenatal care was associated with the level of antenatal care in the neighboring community as an indicator of social norms [39]. Social support is adjacent to the explicit modelled practices of a personal advisors as was measured in this study, however. More parallel exposure measures were used in a 2014 paper by Kumar et al. [36] which found that the number of friends or family members who had gotten vaccinated was a significant predictor of a person getting a vaccine themselves. Acceptability of the HPV vaccine was also found to be highest when “when people believed that important others wanted them to be vaccinated or held favorable beliefs toward the vaccine” [40]. Social network theory as applied to public health is a means by which knowledge of and attitudes towards a health behavior can be formed [41]. In this case, the use of bed nets may be influenced by the individuals whom the pregnant women seek advice from during their pregnancy.

The similar AUC values and odds ratio trends seen in the models using only the number of advisors using nets suggest that this effect is independent of the reported amount of communication with the advisors about malaria and how often the advisors’ advice is perceived to be followed. Despite these results, the modest AUC values of the ownership models suggest that there are strong competing factors other than interpersonal influence on ownership of a bed net. These could be things such as availability, educational level, cost, and perceived risk, which, as noted previously, have been associated with bed net ownership in other studies and may have been responsible for the higher AUC values in the adjusted models [7, 16, 17, 28]. The larger AUC values for the net use models suggest that once owned, the decision to use a net may be more dependent on interpersonal influence.

## Limitations

This study has several limitations. The sample size is relatively small, with only 67 women not owning bed nets and 90 women not using bed nets. This opens the possibility that some of the analyses could have been underpowered; given the conditions of the study, an OR of 2.45 for net ownership and 2.39 for net use were the minimum effect sizes that could be distinguished from chance [42]. While lower effect sizes would not be reliably estimated, this does not diminish the association that was determined between advisor bed net use and the bed net use by pregnant women in this study. The study is also limited by the nature of measures used to determine the social influence, though it nonetheless, provides strong evidence that more studies should be conducted to examine the relationship between social influence and bed net ownership and use. The overall generalizability of these results to all pregnant women is limited by recruitment at an antenatal clinic. Reported rates of bed net use and ownership in this survey are very similar to what was determined in the community-based DHS survey of 2014 in the broader Ashanti region in which household level mosquito net ownership was 71 and 44% of pregnant women reported sleeping under any mosquito net the night before survey. Those individuals who access health care may over-represent women who are further in their pregnancies or women at a higher education or income level, although the 2014 DHS data indicate that in the Ashanti region 98.8% of women sought antenatal care [7]. In addition, a residual concern exists with the accuracy of self-reported bed net usage, though an attempt was made to address this in the sensitivity analyses using responses from multiple bed net use questions and results were consistent.

The findings of this study would be useful in the context of efforts to increase bed net usage, as they suggest that each additional person who uses a bed net has a positive effect on the usage of people around them. This has implications for the planning and modelling of intervention strategies and any context where the marginal benefits of bed net distribution and education are being considered.

## Conclusions

The results of this study suggest that there is an association between the decision by pregnant women to use or own bed nets and the use of bed nets by the people they go to for pregnancy advice in Kumasi, Ghana. Although the cross-sectional design of this investigation prevents the establishment of a causal relationship, similar findings in other disease prevention settings suggest that interpersonal influence could be an important factor in the uptake of bed net use. Further research into the relationship between social influence and bed net use is warranted, as interventions could capitalize on interpersonal relationships to raise bed net ownership and usage rates in Ghana and worldwide.

## Additional files

**Additional file 1.** Main ownership regression model.

**Additional file 2.** Main use regression model.

**Additional file 3.** Sensitivity analysis models.

## Abbreviations

SD: standard deviation
OR: odds ratio
CI: confidence interval
LLIN: long-lasting insecticidal nets
PMI: President’s Malaria Initiative
KSH: Kumasi South Hospital
WHO: World Health Organization
AUC: Area Under the Receiver Operating Characteristic Curve
JSS: Junior Secondary School
JHS: Junior High School
SSS: Senior Secondary School
SHS: Senior High School
