# Supplementary material for: Reported bed net ownership and use in social contacts is associated with uptake of bed nets for malaria prevention in pregnant women in Ghana
Source: Malar J. 2017 Jan 4;16:13. doi: 10.1186/s12936-016-1660-4 (PMC5210303; doi:10.1186/s12936-016-1660-4)
Supplement: Supplementary file 1 — Additional file 1. Main ownership regression model. [file 12936_2016_1660_MOESM1_ESM.docx]

**Additional file 1: Main Ownership Regression Model**

Model of Influence Score^a^ Category versus Bed Net Ownership

|  | **Crude Model** | | | **Adjusted A^b^** | | | **Adjusted B^c^** | | |
| --- | --- | --- | --- | --- | --- | --- | --- | --- | --- |
| **Parameter** | **Estimate** | **95% Confidence Limits** | | **Estimate** | **95% Confidence Limits** | | **Estimate** | **95% Confidence Limits** | |
| Intercept | 1.2462 | 0.9505 | 1.5419 | 1.2463 | 0.7674 | 1.7252 | 1.0753 | 0.2049 | 1.9457 |
| Influence Category (<1 SD Below the Mean) | -0.1815 | -0.5980 | 0.2351 | -0.3032 | -0.7496 | 0.1431 | -0.2365 | -0.6963 | 0.2233 |
| Influence Category (<1 SD Above the Mean) | 0.4121 | -0.1152 | 0.9393 | 0.4687 | -0.0753 | 1.0128 | 0.4995 | -0.0632 | 1.0622 |
| Influence Category (>1 SD Above the Mean) | 0.2579 | -0.3097 | 0.8255 | 0.3738 | -0.2229 | 0.9705 | 0.2997 | -0.3142 | 0.9135 |
| Age 23-27 years |  |  |  | -0.6069 | -1.1054 | -0.1084 | -0.5542 | -1.0736 | -0.0348 |
| Age 27-32 years |  |  |  | 0.4829 | -0.0665 | 1.0322 | 0.4898 | -0.0796 | 1.0592 |
| Age >31 years |  |  |  | 0.0133 | -0.5389 | 0.5655 | -0.0536 | -0.6275 | 0.5204 |
| Marital Status (Married) |  |  |  | 0.0301 | -0.6079 | 0.6681 | -0.0338 | -0.3634 | 0.2957 |
| Educational Level (Junior School) |  |  |  | -0.5940 | -1.0454 | -0.1426 | -0.6173 | -1.0862 | -0.1485 |
| Educational Level (Senior School or More) |  |  |  | 0.2023 | -0.1813 | 0.5858 | 0.2066 | -0.1912 | 0.6045 |
| Has Heard of Malaria in Past Year |  |  |  |  |  |  | -0.1937 | -0.5384 | 0.1510 |
| Believes It Is Easy to Get a Bed Net while Pregnant |  |  |  |  |  |  | -0.2607 | -0.5785 | 0.0572 |
| Has Heard of Using Nets to Prevent Malaria |  |  |  |  |  |  | 0.2040 | -0.5143 | 0.9224 |
| Is Worried About Malaria |  |  |  |  |  |  | -0.2361 | -0.5598 | 0.0877 |
| Knows of a Someone Who Died of Malaria while Pregnant |  |  |  |  |  |  | -0.3266 | -0.8169 | 0.1636 |
| One Advisor or More Got Malaria while Pregnant |  |  |  |  |  |  | 0.4321 | 0.0461 | 0.8180 |
| ^a^Influence Score $=\sum_{j=1}^{j} R_{j}*\left( U_{j}+T_{j} \right)*I_{j})$  ^b^Adjusted for age, marital status, and education level  ^c^Adjusted for age, marital status, education level, and malaria perceptions and attitudes | | | | | | | | | |
