# Supplementary material for: Reported bed net ownership and use in social contacts is associated with uptake of bed nets for malaria prevention in pregnant women in Ghana
Source: Malar J. 2017 Jan 4;16:13. doi: 10.1186/s12936-016-1660-4 (PMC5210303; doi:10.1186/s12936-016-1660-4)
Supplement: Supplementary file 2 — Additional file 2. Main use regression model. [file 12936_2016_1660_MOESM2_ESM.docx]

**Additional file 2: Main Use Regression Model**

Model of Influence Score^a^ Category versus Bed Net Use

|  | **Crude Model** | | | **Adjusted A** | | | **Adjusted B** | | |
| --- | --- | --- | --- | --- | --- | --- | --- | --- | --- |
| **Parameter** | **Estimate** | **95% Confidence Limits** | | **Estimate** | **95% Confidence Limits** | | **Estimate** | **95% Confidence Limits** | |
| Intercept | 0.3677 | 0.0708 | 0.6646 | 0.3159 | -0.1817 | 0.8136 | -0.0154 | -0.8982 | 0.8674 |
| Influence Category (<1 SD Below the Mean) | 0.0572 | -0.3689 | 0.4833 | -0.0495 | -0.5026 | 0.4036 | -0.0566 | -0.5313 | 0.4181 |
| Influence Category (<1 SD Above the Mean) | 0.1857 | -0.2830 | 0.6544 | 0.1991 | -0.2823 | 0.6805 | 0.1354 | -0.3604 | 0.6311 |
| Influence Category (>1 SD Above the Mean) | 0.7309 | 0.1644 | 1.2975 | 0.7542 | 0.1669 | 1.3415 | 0.8014 | 0.1898 | 1.4131 |
| Age 23-27 years |  |  |  | -0.2852 | -0.8288 | 0.2585 | -0.1919 | -0.7642 | 0.3803 |
| Age 27-32 years |  |  |  | 0.0383 | -0.4369 | 0.5135 | 0.0535 | -0.4401 | 0.5472 |
| Age >31 years |  |  |  | 0.2294 | -0.3049 | 0.7637 | 0.1107 | -0.4476 | 0.6689 |
| Marital Status (Married) |  |  |  | 0.3830 | -0.2611 | 1.0271 | -0.2479 | -0.5834 | 0.0875 |
| Educational Level (Junior School) |  |  |  | 0.4052 | -0.1386 | 0.9489 | 0.4199 | -0.1349 | 0.9747 |
| Educational Level (Senior School or More) |  |  |  | -0.2629 | -0.6599 | 0.1341 | -0.2677 | -0.6779 | 0.1424 |
| Has Heard of Malaria in Past Year |  |  |  |  |  |  | -0.2636 | -0.6491 | 0.1220 |
| Believes It Is Easy to Get a Bed Net while Pregnant |  |  |  |  |  |  | -0.2766 | -0.6233 | 0.0700 |
| Has Heard of Using Nets to Prevent Malaria |  |  |  |  |  |  | 0.2050 | -0.5350 | 0.9450 |
| Is Worried About Malaria |  |  |  |  |  |  | -0.1542 | -0.5031 | 0.1947 |
| Knows of a Someone Who Died of Malaria while Pregnant |  |  |  |  |  |  | 0.0294 | -0.3899 | 0.4487 |
| One Advisor or More Got Malaria while Pregnant |  |  |  |  |  |  | 0.4158 | -0.0277 | 0.8594 |
| ^a^Influence Score $=\sum_{j=1}^{j} R_{j}*\left( U_{j}+T_{j} \right)*I_{j})$  ^b^Adjusted for age, marital status, and education level  ^c^Adjusted for age, marital status, education level, and malaria perceptions and attitudes | | | | | | | | | |
