# Supplementary material for: Reported bed net ownership and use in social contacts is associated with uptake of bed nets for malaria prevention in pregnant women in Ghana
Source: Malar J. 2017 Jan 4;16:13. doi: 10.1186/s12936-016-1660-4 (PMC5210303; doi:10.1186/s12936-016-1660-4)
Supplement: Supplementary file 3 — Additional file 3. Sensitivity analysis models. [file 12936_2016_1660_MOESM3_ESM.docx]

**Additional file 3: Sensitivity Analysis Models:**Adjusted^a^ Odds Ratios of Self-Reported Bed Net Ownership and Use for Alternative Influence Scores

|  | **Bed Net Ownership (n=294)** | | | | | | | |
| --- | --- | --- | --- | --- | --- | --- | --- | --- |
|  |  | | | | | | | |
| **Category** | Alternative Influence Score^b^  OR (95% CI) | Following of Advice Score^c^  OR (95% CI) |  |  | | **Number of Advisors** | | Number of Advisors Talked to About Malaria |
|  |  |  |  |  | |  | |  |
| **>1 SD below mean** | Reference | Reference |  |  | | **0** | | Reference |
| **0-1 SD below mean** | 1.30 (0.56, 3.04) | 0.97 (0.43, 2.18) |  |  | | **1** | | 1.25 (0.56, 2.78) |
| **0-1 SD above mean** | 1.86 (0.77, 4.46) | 1.02 (0.46, 2.24) |  |  | | **2** | | 0.64 (0.26, 1.60) |
| **>1 SD above mean** | 2.15 (0.82, 5.64) | 0.78 (0.36, 1.75) |  |  | | **3** | | 0.56 (0.25, 1.22) |
|  |  |  |  |  | |  | |  |
| **Model AUC** | 0.721 | 0.634 |  |  | | **Model AUC** | | 0.706 |
|  | **Bed Net Use (n=227)** | | | | | | | |
| Influence Score^a^ | Alternative Influence Score^a^  OR (95% CI) | Following of Advice Score^b^  OR (95% CI) |  | | Number of Advisors | | Number of Advisors Talked to About Malaria  OR (95% CI) | |
|  |  |  |  |  | |  | |  |
| **>1 SD below mean** | Reference | Reference |  |  | | **0** | | Reference |
| **0-1 SD below mean** | 2.57 (1.03, 6.44) | 1.83 (0.77, 4.33) |  |  | | **1** | | 0.99 (0.49, 2.02) |
| **0-1 SD above mean** | 5.76 (2.13, 15.57) | 0.67 (0.32, 1.42) |  |  | | **2** | | 1.17 (0.43, 2.91) |
| **>1 SD above mean** | 3.71 (1.49, 9.23) | 0.70 (0.31, 1.54 |  |  | | **3** | | 1.08 (0.48, 2.38) |
|  |  |  |  |  | |  | |  |
| **Model AUC** | 0.727 | 0.649 |  |  | | **Model AUC** | | 0.656 |
| ^a^ Adjusted for age, marital status, education level, and malaria perceptions and attitudes  ^b^Alternative Influence Score $=\sum_{j=1}^{j} \left( U_{j}+T_{J} \right)*I_{J})$  ^c^_­_Following of Advice Score= $=\sum_{j=1}^{j} \left( I_{j} \right)$ | | | | | | | | |
